# The Role of Physical Exercise in Opioid Substitution Therapy: Mechanisms of Sequential Effects

**DOI:** 10.3390/ijms24054763

**Published:** 2023-03-01

**Authors:** Alexandros Psarianos, Costas Chryssanthopoulos, Thomas Paparrigopoulos, Anastassios Philippou

**Affiliations:** 1 1st Department of Psychiatry, Medical School, National and Kapodistrian University of Athens, 11528 Athens, Greece; 2 Department of Physiology, Medical School, National and Kapodistrian University of Athens, 11527 Athens, Greece

**Keywords:** opioid maintenance treatment, opioid users, exercise in opioid substitution treatment, exercise-induced mechanisms of sequential effects

## Abstract

It is generally accepted that chronic opioid use is associated with structural and functional changes in the human brain that lead to an enhancement of impulsive behavior for immediate satisfaction. Interestingly, in recent years, physical exercise interventions have been used as an adjunctive treatment for patients with opioid use disorders (OUDs). Indeed, exercise has positive effects on both the biological and psychosocial basis of addiction, modifying neural circuits such as the reward, inhibition, and stress systems, and thus causing behavioral changes. This review focuses on the possible mechanisms that contribute to the beneficial effects of exercise on the treatment of OUDs, with emphasis placed on the description of a sequential consolidation of these mechanisms. Exercise is thought to act initially as a factor of internal activation and self-regulation and eventually as a factor of commitment. This approach suggests a sequential (temporal) consolidation of the functions of exercise in favor of gradual disengagement from addiction. Particularly, the sequence in which the exercise-induced mechanisms are consolidated follows the pattern of internal activation—self-regulation—commitment, eventually resulting in stimulation of the endocannabinoid and endogenous opioid systems. Additionally, this is accompanied by modification of molecular and behavioral aspects of opioid addiction. Overall, the neurobiological actions of exercise in combination with certain psychological mechanisms appear to promote its beneficial effects. Given the positive effects of exercise on both physical and mental health, exercise prescription is recommended as a complement to conventional therapy for patients on opioid maintenance treatment.

## 1. Introduction

We are witnessing an opioid use epidemic outburst [1], which, due to the COVID-19 pandemic, is negatively fed [2,3], with a steadily increasing trend [4,5,6] and characterized by frequent overdose events [4,6,7] and relapses [8]. Indeed, even though people have learned enough to be able to make a correct diagnosis and use a variety of pharmaceutical and psychotherapeutic treatments [9], relapses [10] and overdoses [11] happen often [7,8]. The extensive literature suggests that opioid use causes a wide range of side effects [12] and is closely associated with individuals’ low interest in physical exercise [13], which results in low levels of physical activity [13,14,15]. A passive way of dealing with everyday life is adopted, which often acts at the expense of extroversion. Abstaining from various pleasurable activities due to their passive way of life deprives these people of beneficial positive interaction [16,17]. Furthermore, opioid use is associated with a significant reduction in quality of life, given that the basic activities of daily living are replaced by the constant search for or use of the substance [18]. At the same time, stigma, prejudice, and fear further marginalize addicts, leading to even greater isolation. This marginalization can cause depressive responses, anxiety, and low self-esteem, which help maintain addiction [19]. 

Treatment by using substitute substances (methadone and buprenorphine) is currently the mainstay of treatment for opioid addiction [20,21], as it is safe and effective in suppressing illicit opioid use [2,22,23,24,25]. It helps improve physical and mental health [26,27] as well as reduce mortality, especially death from overdose [22,28,29]. However, like all opioids, methadone (a complete opioid receptor agonist) and buprenorphine (a partial opioid agonist) are also addictive, and there is a possibility of causing side effects when they interact with other drugs [23,30]. These substances are also linked to diabetes and high levels of nicotine addiction [31,32]. Nearly 90% of participants in opioid substitution programs smoke cigarettes, increasing their cardiovascular risk [33,34,35,36]. Yet, people receiving maintenance therapy (methadone and buprenorphine) often experience physical and psychological symptoms that require further health care [37]. Specifically, osteoporosis [38,39], musculoskeletal pain [40], disproportionate weight gain [41,42], increased risk of developing hypertension and coronary heart disease, sleep disorders [43], and mood disorders [13,44] are common disorders. In addition, compared to the perception of the general population, they perceive their health as worse [45]. At the same time, comorbidity (dual disorder) [46] is a common phenomenon in this population, with high rates of anxiety and depression [29]. Clinical studies show that about half of the population receiving methadone and buprenorphine maintenance therapy will have depression at some point in their lives, while nearly one-third report a depressive mood on treatment [47]. 

Opioid addiction is a multifactorial disorder with a biopsychosocial etiology [48,49]. It is a chronic disorder characterized by relapses and remissions [8,50], triggered by biological, psychological, and social factors [51]. It is determined by a complex of cognitive, behavioral, and physiological symptoms, which include: (a) a strong desire (craving) or urge to take the substance; (b) difficulties or unsuccessful attempts to stop or control the use; (c) withdrawal syndrome; (d) the development of tolerance; (e) the progressive neglect of alternative interests due to substance use; and (f) persistent use in circumstances that are physically and psychologically dangerous [52,53,54]. It also leads to morphological and functional alterations of various brain structures, especially the reward system, through a multifaceted network of interconnected structures. This structure consists of serotonergic, cannabinergic, opioidergic, GABAergic, glutamatergic, and dopaminergic systems. This also regulates the total release of dopamine [55,56] in many regions of the nucleus accumbens (NAc) involved in memory, decision-making, pleasure and anxiety [57,58,59]. Additionally, this makes NAc a homeostatic reward factor that controls urges and desires. The prefrontal cortex, which moderates these drives and desires depending on experience, learning, and long-term behavioral goals, is also affected [60]. A key factor in this process is the endocannabinoid (eCB) system, a widely distributed neuromodulatory network that regulates synaptic excitability and the release of neurotransmitters through its two main endogenous ligands, N-arachidonoylathanolamine (AEA) and 2-arachidonoyl glycerol (2-AG). It has been found that the eCB system is impaired in patients with a substance use disorder (SUD) [21,22], which may contribute to increased mood disorders and increased stress reactivity [61,62]. Pharmacological targeting of the eCB system has resulted in the improvement of SUD treatment outcomes in both preclinical and clinical studies [63,64]. Other effective treatment options are currently available [19,65,66,67], but relapses at some point are common [68].

There is considerable research-based evidence that patients with opioid use disorders’ participation in organized exercise programs result in significant beneficial effects [50,68,69,70,71,72,73,74,75,76,77,78]. This is while physical activity has positively affected opioid users on maintenance therapy [50,68,69,70,76,77]. In particular, physical activity is any movement that makes you use more energy. This is different from exercise, which is a planned, structured, repetitive, and intentional physical activity to improve or maintain physical fitness [79]. Furthermore, this improvement is observed at the level of both physical and mental health, while substance use is also reduced [13,70,80,81,82,83,84]. More specifically, there is the restoration of bone quality [85], reduction of musculoskeletal pain [86], improved levels of high (HDL) and low (LDL) density lipoproteins, triglycerides (TG), and fibrinogen [87], maintenance of healthy body weight [88], and an overall improvement of physical condition (heart rhythm, muscle mass, blood pressure) [82,84]. Improving sleep quality [69,89,90], reducing perceived stress [44] and symptoms of depression [91], regulating stress levels [92], and overall improving quality of life [13,69,82,83] are all ways to improve mental health. Consequently, treatment compliance is enhanced [70,82,91], resulting in a reduction in substance use [93]. Physical exercise is an appealing, healthy, and substitute behavior in patients with opioid use disorders due to the aforementioned positive effects. 

Overall, both interventional and observational studies showed that the activity status in patients with opioid use disorders (OUDs) is low, with almost no high-intensity exercise load, while the most significant health benefits were observed in long-duration training programs of moderate or higher intensity [70]. Moreover, different types of voluntary exercise such as yoga, weight lifting, treadmill running, swimming, indoor cycling, and stationary cycling, have been used to treat substance abuse disorders [94,95,96]. Aerobic exercise, on the other hand, has been shown to help treat stressors like long-term drug use, with therapeutic effects similar to those of drug or talk therapy for people with OUDs [97].

This specific form of exercise (aerobic) has beneficial effects on the physiological mechanisms that regulate the symptoms of addiction [69,71,91,92]. It activates and modifies several neural circuits (i.e., reward, inhibition, and stress circuits) [75,92,98] through a network of structures and systems that communicate with each other. This includes the endogenous opioid [91] and endocannabinoid systems [99], which can act as a mood enhancer and help reduce the desire for drug use [69,71,100,101,102]. Specifically, exercise can be a non-pharmacological intervention for enhancing the eCB system due to increasing the circulating levels of endocannabinoids in healthy individuals [103,104,105]. This is because it can activate the eCB system in patients with SUD, leading to mood improvements, better stress management, and attenuated withdrawal symptoms [63,64]. Concerning the psychological symptoms of abuse and dependence, physical exercise may serve as an alternative strategy for dealing with such symptoms [83,106,107]. 

**Figure 1:**
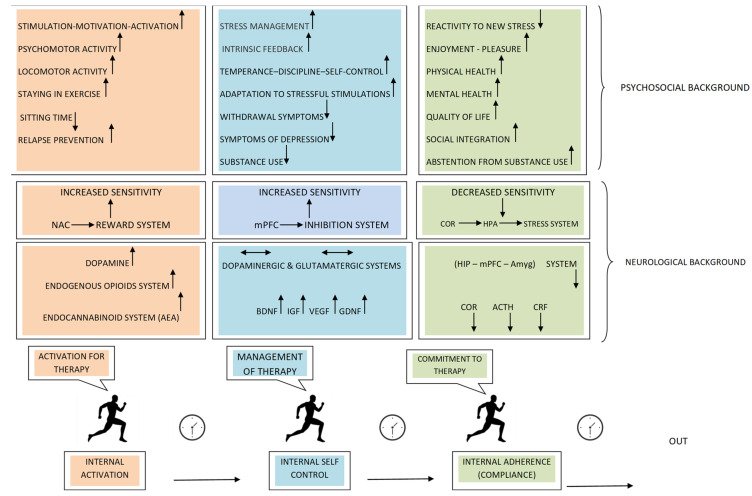
Physical exercise has beneficial effects on both the biological and psychosocial underpinnings of addiction, acting initially as a factor of internal activation and management and eventually as a factor of commitment and adherence to therapy. This approach suggests a sequential (temporal) consolidation of the functions of exercise in favor of gradual disengagement from addiction. The sequence of events follows the pattern of Internal activation—Self-regulation—Commitment, eventually resulting in the modification of molecular and behavioral aspects of opioid addiction. Physical exercise has the following effects: no change/balance, upregulation, and downregulation.

In this review, a novel approach is suggested regarding the role of exercise in opioid substitution therapy. Exercise’s functions are described as a sequential consolidation that leads to gradual disengagement from addiction. Indeed, physical exercise exerts its actions sequentially. It first acts as an internal activator by affecting the endogenous opioid and cannabinoid systems and boosting dopaminergic transmission [91]. Then, acts as a self-regulating and modulating factor by regulating dopaminergic and glutamatergic neurotransmission at the level of neurotransmitters, receptors, and transporters and by reversing drug-induced molecular damage in the medial prefrontal cortex [92]. Finally, it acts as a factor in commitment to therapy due to its known benefits for brain health, sleep, overall quality of life, and reduction of anxiety and depression [69] (Figure 1). This approach opens a new perspective on the therapeutic framework of opioid substitution programs, which might be greatly enhanced by the utilization of physical exercise [108]. Therefore, further research in this area is considered necessary and promising.

## 2. Role of Internal Activation—Self-Regulation—Commitment in Opioid Substitution Programs 

Internal mobilization activation. This factor intensifies the individual’s effort, increases perseverance, and essentially potentiates it. Actions directed by inner motivation are manifested for the pleasure and satisfaction derived from their accomplishment and are not intended for external reward. The person who tries to achieve this therapeutic goal tries to have full physical and mental capacity. Success based on psychosomatic competency increases self-esteem and feelings of competence, which in turn feedback on motivation [109,110,111].

Self-regulation Management. With self-regulation, the external regulations are internalized, better understood, and identified with the values and needs of the individual. People who exercise tend to have a better mood and higher activity levels for several hours after exercise. People with satisfactory levels of physical fitness better regulate physiological mechanisms, such as muscle aches and tension, heart rate, and blood pressure, and respond better to stressful situations than people who do not exercise [112,113].

Commitment and Dedication. High levels of activation, a sense of achievement, and positive self-feelings contribute to a reduced response to stressful stimuli [114] and the develop a pleasant framework [115,116]. This inspires individuals to maintain a consistent commitment and dedication to their exercise efforts [50,117]. In addition, it generally affects people’s lifestyles who also embrace a variety of other similar health behaviors [118,119]. 

## 3. The Role of Temporal Placement (Serialization) of the Sequence of Internal Activation—Self-Regulation—Commitment in Opioid Substitution Programs

The opioid substitution treatment programs consist of a phasic process, characterized by remissions and relapses, which may be constantly modified according to the needs and the clinical course of the patient. Thus, treatment strategies should be applied as needed, and physical exercise should be scheduled accordingly to serve the scope, taking into account the different mechanisms of exercise actions [120]. These mechanisms are consolidated in a temporal sequence (internal activation—self-regulation—commitment) and modify molecular and behavioral parameters related to opioid dependence.

## 4. Mechanisms of Sequential Effects of Exercise in Opioid Substitution Therapy—Biological Background

It is widely accepted that the benefits of exercise on physical and mental health are the result of the activation of long-term and sequential biological mechanisms [121,122]. For example, exercise reduces the incidence of obesity-associated diseases [121,122]. Additionally, it improves learning by increasing the levels of the brain-derived neurotrophic factor (BDNF), which in turn contributes to neurogenesis, angiogenesis, and the development of new dendritic branching in brain regions that are critical for learning and memory [123,124,125,126,127]. 

In OUDs, physical exercise appears to be a non-pharmacological treatment [69,71,72], acting through the release of endocannabinoids [99,128] and endogenous opiates [91,92,129], mobilizing the same neural systems in the brain that are also involved in the addiction process [73,130]. Although cannabinoids and opioids are different classes of drugs, activation of the (endo)cannabinoid and opioid systems has similar effects, and molecular and pharmacological studies support the existence of reciprocal interactions between the two systems. This suggests a common underlying mechanism [131]. More specifically, the eCB system plays a key role in maintaining homeostasis, which includes the regulation of metabolism and stress responses. Chronic stress can blunt eCB signaling, and disturbances in the eCB signaling have been linked to stress-related psychiatric disorders and physical health conditions, such as anxiety, depression, posttraumatic stress disorder (PTSD), diabetes, and obesity. Targeting the eCB system with pharmacological and non-pharmacological behavioral interventions, such as physical exercise, appears to be a promising therapeutic approach for the prevention and treatment of stress-related diseases [99]. Given that exercise is a potent activator of the eCB system [132], it could be a particularly effective intervention for reducing psychosomatic withdrawal, managing stress, and alleviating drug cravings [128,132]. Physical exercise has also been shown to have a buffering effect on neurotransmission by regulating dopaminergic and glutamatergic neurotransmission at the level of neurotransmitters, receptors, and transporters and reversing drug-induced molecular damage caused by chronic psychoactive ssubstance use [91,92]. Moreover, physical exercise may facilitate abstinence by ameliorating low mood via effects on the endogenous opioid system and the potentiation of dopaminergic transmission [91]. In addition, prolonged periods of aerobic exercise may reduce opioid self-administration by modifying the number of central opioid receptors [133] while it also helps reduce anxiety and depression and improves the overall quality of life of patients with OUD [69]. Based on these findings, the prevailing view is that physical exercise can be an effective alternative for addictive substances [98,100], improve retention in opioid agonist treatment [72], and play a positive role in opioid addiction recovery [71]. 

However, despite the considerable progress in our understanding of the impact of physical exercise on substance abuse prevention and treatment [98,134,135], there is little evidence regarding the neurobiological mechanisms that mediate the relationship between physical exercise and drug abuse vulnerability. At the same time, exercise-induced changes in reward-related neural systems are potentially considerable [69,98]. Nevertheless, there is no evidence that these changes may be mediated by a mechanistic model of sequential exercise-induced effects on neurobehavioral processes such as impulsivity, emotion regulation, stress, and executive and cognitive function. The efficacy of this model may be attributed to its competence to make easier dopaminergic transmission (reward system) [91], later to its ability to regulate glutamatergic and dopaminergic signaling in the medial prefrontal cortex (mPFC) and to reverse drug-induced changes in chromatin through epigenetic interactions with BDNF (inhibition system) [92]. Finally, this model’s efficacy can be attributed to its ability to reduce cortisol levels as well as the Hypothalamic-Pituitary-Adrenal (HPA) axis responses to new stress (stress system) [136] and improve treatment retention and overall quality of life in OUD patients [69] (Figure 1). 

Overall, physical exercise can prevent the development of addictive behaviors, suppress the desire for the substance, and contribute effectively to detoxification [69,72]. In particular, addictive substances can cause structural and functional alterations in the brain at the cellular and molecular levels. Physical exercise can modify the extent of these changes as it has a common neural circuitry with addictive substances [73,92], creating a new stimulating environment that evokes emotions related to those of substance abuse, thus making their deficit less significant, while it blocks the neuro transformations in the anterior prefrontal cortex and mitigates the drug-seeking behaviors [137]. 

### 4.1. Exercise as an Internal Activation Factor 

Opioid use disorders are traditionally associated with rapid and sharp fluctuations in extracellular dopamine concentration and sensitiveness in the brain’s reward system [55]. This neural network consists of many communicating regions in the brain, including the ventral capillary region of NAc, the amygdala, the striatum, the hippocampus, and the prefrontal cortex (RFC) [138,139]. Some of these neural network components that are important for addiction vulnerability may be modified by physical exercise. Specifically, systematic physical exercise increases the sensitivity of NAc function, as it is considered a neural interface between motivation and action, possessing a key role in reward-motivated behavior, stress-related behavior, and substance dependence [140], eventually increasing the reward sensitivity [101]. 

More specifically, physical exercise activates the dopaminergic system [91,92], which plays a central role in the process of addiction [141]. The new transmitters that occur during exercise mediate buffering effects at all three levels of dopaminergic biomarkers (neurotransmitter, receptor, and transporter level), such as increased plasticity, dopamine synthesis, and postsynaptic D2 receptors, decreased presynaptic D2 receptors, as well as other alterations in the regulatory dopaminergic system [91,92]. At the behavioral level, the release of dopamine into areas of the midbrain can lead to a modification of the emotional mediation of deterrent stimuli [74]. Thus, at the beginning of substance use, physical exercise can act as an alternative enhancer (exerting a protective effect) by increasing dopaminergic signaling. In addition, through the neuroadapters mentioned above, moderate-intensity exercise can reduce susceptibility to substance use, while high-intensity exercise, mimicking the action of substances, can increase the vulnerability to substance use. Finally, exercise can exert a protective effect against addiction by normalizing the alterations in dopamine levels that occur in the substance-use deprivation cycle [139,142]. However, the mesocorticolimbic dopamine pathways do not work alone. As has already been mentioned, they are part of a multifaceted network of interconnected structures consisting of serotonergic, cannabinoid, opioidergic, GABAergic, glutamatergic, and dopaminergic systems, each of which plays a unique role in reward-seeking [143]. These cascading interactions lead to a balanced dopamine release in many effector-specific regions of the NAc involved in memory, decision-making, pleasure, and anxiety [57,58,59,144]. In recent decades, there has been significant progress in the understanding of the actions of cannabinoids and the endocannabinoid system in reward processing and the development of addictive behavior. Cannabis-derived psychoactive compounds, such as Δ9-tetrahydrocannabinol and synthetic cannabinoids, interact directly with the reward system and, therefore, have addictive properties. Cannabinoids exert their reinforcing properties by increasing tonic dopamine levels via a cannabinoid receptor type 1 (CB 1)-dependent mechanism within the ventral tegmental area. Cues that depend on cannabis smoking can cause drug-seeking behavior (i.e., craving) by triggering phasic dopamine events [145]. Interestingly, exercise-induced mobilization of eCBs contributes to the replenishment of energy stores and mediates various mood-elevating analgesic effects. Indeed, acute increases in the levels of AEA (eCBs) in circulation have been reported after exercise, documenting an exercise-induced activation of the eCB system in both patients with SUD and healthy subjects [132]. More specifically, in healthy individuals, elevated levels of AEA after exercise have been associated with many beneficial psychological effects, including positive emotion and courage, as well as reduced reactivity to stressful stimuli [105]. Exercise-induced AEA increases can restore the eCB system, which is often downregulated due to the chronic use of addictive substances [146]. This leads to inadequate and abnormal stress responses [147]. Overall, exercise through the activation of the eCB system can exert positive effects in the prevention and treatment of stress-related psychopathology [99].

Additionally, it is well known that endogenous opioids affect the brain’s reward system by releasing potent neurotransmitters that cause feelings of euphoria [148]. Exercise has the potential to increase circulatory concentrations of endogenous opioid peptides [91,130], which are bound to all three major opioid receptor types (mu, kappa, and delta) [149,150,151,152,153]. During exercise, especially during the first 20 min, several endogenous opioids are released [154], leading to the enhancement of behavior and, thus, to its recurrence [153]. Endogenous opioids have pharmacological properties similar to those of opiates such as heroin and morphine [148], potentially making exercise a compatible substitute for drug use [91]. In addition, prolonged periods of aerobic exercise cause alterations in opioid-binding proteins [155] and reduce susceptibility to exogenously administered opioid agonists [156]. The release of endogenous opioid peptides through exercise may induce a calming effect on individuals, both psychologically and physically [69,91,135]. 

Overall, systematic exercise acting via the endogenous opioid system and enhancing dopaminergic transmission [91] increases the sensitivity of NAc function and results in increased reward sensitivity [101]. These changes mobilize the individual, creating an internal stimulating environment that increases self-esteem and feelings of adequacy, thereby increasing motivation for effort [110,111], (Figure 1). 

### 4.2. Exercise as a Self-Regulation and Management Factor

Studies using position emission tomography (PET) and functional magnetic resonance imaging (fMRI) have shown that individuals with SUD have reduced activity in the mPFC. The mPFC is widely accepted to contribute to several addiction-connected operations, including reward sensitivity, inhibitory control, stress reactivity, and emotion regulation [157,158]. This state appears to be associated with a diminished number of dopamine receptors as well as an abnormal rate of dopaminergic neuron firing [159]. These alterations in the dopamine system and PFC activity may lead to an impulsive and forced desire for the substance, as well as a loss of self-control with its intake [158]. Similarly, incomplete development of the prefrontal cortex and the resulting reduction in adequate control of impulsive decisions leads to an increased risk of developing addictive behavior and has been suggested as an explanation for drink abuse [160]. 

Structural and functional changes in mPFC neurons can be induced by exercise, increasing the sensitivity of mPFC function, as reflected by an increase in inhibitory control, executive and cognitive function, as well as emotion regulation [92,101]. More specifically, structured aerobic exercise programs can provide improved intrinsic neuromuscular feedback, leading to higher levels of neurotrophic factors (BDNF, Glial cell line-derived neurotrophic factor-GDNF), insulin-like growth factor 1-IGF-1, and vascular endothelial growth factor-VEGF). The neurotrophic factor BDNF is an intracellular signaling molecule, the concentrations of which increase in the human brain with aerobic exercise. The increase in BDNF concentrations is likely to normalize the changes in neuronal synapses caused by repeated administration of substances, exerting a protective effect, especially in those areas that are responsible for drug-seeking behavior [161]. VEGF production is also enhanced by exercise, leading to an increase in blood vessels in the hippocampus, cortex, and cerebellum [162,163]. 

Exercise also appears to increase IGF-1 levels in the circulation and the hippocampus, although data from human studies are conflicting [164,165]. Other intracellular signaling molecules, such as PKA (cAMP-protein kinase A), DARPP-32 (dopamine cAMP-regulated neuronal phosphoprotein), and ERKs (extracellular signal-regulated kinases), which act as mediators in seeking addictive substances, are also positively affected by aerobic exercise [129,166]. Aerobic exercise has also been shown to cause neurogenesis in the hippocampus [167], and gliogenesis in the prefrontal cortex, both in experimental animals [126] and humans [168]. 

In addition, prolonged physical exercise increased the availability of the dopamine-binding receptor. It is important to note that acute periods of exercise increase dopamine concentrations [135,169], while chronic periods of exercise affect the expression of several dopamine-binding receptors [73,170,171]. Therefore, normalizing the changes in dopamine levels occurs in the substance use-withdrawal cycle [139,142]. These effects of exercise may impair substance-seeking behavior, particularly in individuals with dopamine or neurotrophic deficits [172]. Additionally, they can act as alternative behavior enhancers and ultimately lead to a reduction in the forced taking of the substance (protective action of exercise) [55]. 

Other signaling pathways than dopamine, such as glutamate pathways, are also involved in the biological mechanisms of addiction. They encourage the use of addictive substances, especially during the dependent state of addiction, e.g., when the addiction has developed during relapse [173,174,175,176]. In particular, it is known that glutamate contributes to the incubation of addiction (dependence state) by mediating the process of desiring the substance (craving) [177]. 

Exercise is likely to have a positive effect on glutaminergic system regulation, both directly, normalizing its levels, especially during periods of deprivation, when it is deregulated, and indirectly, regulating elevated dopamine levels [98,139]. This regulation results in positive mood changes and the reduction of stress, as well as changes in muscle tension, heart rate, and blood pressure [113,178]. The normalization of the increased activity of glutaminergic projections from the prefrontal cortex and tonsils to the abdominal cap region and the striatum has been shown to play a regulatory role and prevent the uptake of toxic substances after exposure to environmental stimuli or stress [179]. 

Overall, regular exercise can reduce the desire to use drugs during both relapse and recovery. It accomplishes this by regulating the dopaminergic and glutamatergic systems at every level, including the neurotransmitters, receptors, and transporters [92]. In particular, by normalizing the changes in dopamine levels, which usually occur in the substance use-withdrawal cycle [139,142], as well as glutamate signaling, it can reverse molecular damage caused in the mPFC region [98,180,181]. As a result, physical exercise increases the sensitivity of mPFC function, which is reflected in an increase in inhibitory control, executive function, and cognitive function, as well as emotion regulation [101], eventually acting as a self-regulation factor [182]. 

### 4.3. Exercise as a Factor of Commitment and Adherence (Compliance)

The development of addiction comprises a focal transposition from positive to negative reinforcement, corresponding to neurobiological alterations in stress systems in the brain [157]. These changes lead to increased distress and greater allostatic load [183]. Anxiety is a major feature of this process, and neuroadaptations in the HPA axis lead to gradually greater negative affective states, which eventually result in changes in reward-motivational systems related to substance use [184]. 

Specifically, during severe substance use, the reward system becomes excessively active, causing a rapid and intense fluctuation in the concentration of extracellular dopamine in both the NAc and striatum, which is associated with the feeling of pleasure and euphoria induced by the substance use [185]. However, repeated intake of addictive substances leads to neuroadaptations involving alterations in systems other than those mediating the euphoric effects of the substances. A typical example is the involvement of corticotropin-releasing factor (CRF), the levels of which increase in the amygdala during the withdrawal phase of addictive substances [157,186]. CRF production is initially controlled by the negative feedback of cortisol at the level of the hypothalamus and pituitary gland, however, as the addiction progresses, CRF levels increase excessively in the amygdala during the withdrawal phase. This increase is related to the appearance of irritability, discomfort, emotional loading, and generally anxious behavior [183,185]. 

In individuals with OUDs, there is evidence that opioid administration directly affects the HPA axis and is associated with the suppression of its activity [187,188,189]. Thus, in patients with active opioid use and opioid agonists, including methadone and buprenorphine, cortisol levels are suppressed [190,191,192]. This is while subsequently, their levels increase in response to opioid withdrawal [191,193]. More specifically, opioid withdrawal corresponds to significant stimulatory increases in adrenocorticotropic hormone (ACTH) and cortisol levels, regardless of whether it was induced by naloxone administration [194] or occurred as a natural withdrawal over time [195]. There is evidence that HPA axis dysfunction can be persistent in the inceptive acute stage of withdrawal, with salivary cortisol levels being notably higher in opioid use patients compared with controls for at least 25 days after withdrawal [193]. In turn, increased baseline levels of cortisol due to withdrawal have been associated with anxiety and cognitive impairment [196]. These cognitive disturbances could perpetuate the worsening of addiction [197,198,199]. As a result, dysfunction of HPA activity is observed in patients addicted to opioids, which is associated with either a suppression of cortisol in response to administration or an increase in cortisol levels in response to withdrawal. Similar impaired activity of the HPA axis is also evident in other forms of addiction, such as alcohol [200,201] and smoking [202], and may be accountable for feelings of discomfort and negative reinforcement but also may be associated with various nutritional and metabolic alterations. This results in fatigue, muscle weakness, and an increased risk of infection or related diseases in patients with alcohol use disorder (AUD) [200]. Eventually, HPA axis dysregulation reduces the ability of addicted individuals to experience normal levels of reward, which leaves them more vulnerable to experiencing stress [203]. Hence, stress management is essential for people with substance use disorders, smokers, and patients with AUD. This is because their stress levels are higher [204] and less effectively managed than those of the general population [205], driving them to substance use for relief. Using substances repeatedly activates the stress axes continually, increasing stress and impairing the natural response mechanism [206]. 

The stress system is activated in response to various signals [207], including exercise, which is a type of physical stress that disrupts the body’s homeostasis. To maintain homeostatic balance, the body activates the autonomic nervous system and the HPA axis, resulting in elevated cortisol and catecholamine levels in the circulation [208]. However, regular physical exercise reduces the tendency to increase cortisol levels by recruiting the hippocampus-medial prefrontal cortex-amygdala neurocircuitry [101]. During exercise, cortisol acts as an inhibitor of the hypothalamus and pituitary glands through receptors in the medial prefrontal cortex. It also normalizes the overactivity of the amygdala caused by stress. Thus, a negative feedback system is formed that is tightly regulated by circulating cortisol levels. This circuit restores homeostasis and is indicative of a healthy response to stress [101]. Consequently, addicted individuals who exercise are protected via the mechanism of cortisol reduction, which mitigates their responses to stressful stimuli [101,209] (Figure 1). 

Exercise can improve the regulation of psychological stress by enhancing the communication between the stress axes so that they act more effectively when activated by stress [210]. Indeed, physical exercise has been shown to reduce the individual’s response to stress [211] and may replace substance use as a substitute in unpleasant and painful situations.

Exercise also affects the serotonergic system by increasing the levels of neurotransmitters such as beta-endorphins, epinephrine, norepinephrine, serotonin, and dopamine [151,212]. These are the same neurotransmitters that make exercise rewarding. This may explain why exercise improves stress-related psychiatric disorders, such as depression and anxiety, in people with substance use disorders [129].

Exercise improves the individual’s competence to manage daily stress without using substances [213]. It can also serve as a healthy substitute behavior that maintains individuals’ temperament, character, and self-control by regularly shifting their attention from substance abuse to pleasurable physical and mental habits [214]. Furthermore, exercise-induced satisfaction of the individuals’ three basic needs becomes appealing to them, i.e., autonomy (the need to organize their behavior as they choose and regulate their actions), adequacy (the need to feel effective in what they strive for), and social relationships (the desire for relationships with others, care, and coexistence) [110,215] This satisfaction acts as a reward that strongly activates the midbrain DA system. In turn, the midbrain DA system encodes a reward prediction that makes learning stronger and makes people less sensitive to stressful stimuli [114]. When such a reward is chronic, it can modulate the DA system, which encodes an average rate of reward that influences the dynamics of action and leads to commitment to that action [117,119].

## 5. Mechanisms of Sequential Effects of Exercise in Opioid Substitution Therapy—Psychosocial Background

A large body of evidence demonstrates that opioid use is associated with a high incidence of a wide spectrum of psychiatric disorders, such as anxiety, depression, post-traumatic stress disorder, and personality disorders [46]. Opioid use causes several symptoms, such as not being able to balance and control your emotions [44] and being more sensitive to stress management [13]. This is accompanied by exposure to painful stimuli, thus pushing individuals to re-use substances to relieve themselves [216,217]. It is also related to a significant deterioration in the quality of life as individuals develop automatic behaviors, such as drinking or substance use, during their treatment. This automatic behavior leads to a passive approach to life with overall low physical activity status, as individuals do not have the surplus energy they need [13,14,15], and basic everyday activities are replaced by constant opioid seeking or use [46]. It is also associated with an increased risk of relapse due to individuals’ vulnerability to environmental cues of use that reinforce memories of pleasurable and rewarding outcomes from previous substance use [218]. As a result, extroversion and participation in various pleasant events occur, from which individuals expect to receive positive enforcement [16,17]. 

Engagement in an accessible, immediately rewarding, sustainable, and safe behavior, like physical activity, can reduce relapse in individuals with OUDs by suppressing the individuals’ urge to drink or use the substance. Indeed, physical exercise can serve as an alternative strategy for managing the effects of opioid use disorders [50,70,71,72,83,106,107] because it acts as a component of a healthy lifestyle accompanied by positive behaviors, such as a healthful diet, that are not consistent with alcohol or substance use [69,89,93]. Moreover, adopting regular exercise as part of a healthy lifestyle leads to feelings of energy and motivation, as well as enhanced well-being. This can eventually increase self-efficacy for maintaining abstinence from opioid use [69,81,91]. 

Existing research also supports an inverse relationship between systematic exercise participation and mental health problems in opioid-dependent patients [13,69,70], particularly depression and negative mood [13,82,83], anxiety [44,77], and comorbid mental health disorders [13] that influence the OUD outcomes [50,72,80,84]. More specifically, research has shown significant beneficial effects of exercise on the reduction of symptoms of depression [13,82,83], anxiety [44,77], and acute craving, on mood regulation [91,92], and stress reactivity, as well as on improving group activity and social support. Overall, there is increased compliance with the treatment [72,82], and the visible psychosomatic improvement leads to a reduction in use [93], (Figure 1). 

### 5.1. Exercise as an Internal Activation Factor 

All the aforementioned characteristics of opioid substances are associated with negative mental health conditions, including stress, depression, etc., and impart a feeling of negative mood and an overall malaise to individuals, acting as an inhibiting factor in participating in exercise programs. In particular, patients who receive an opioid agonist and follow passive coping strategies or avoidance strategies are more likely to use substances and have more extensive health problems [78]. They exhibit low interest in participating in the exercise [13], which leads to low physical activity levels [13,14], or physical inactivity [15]. However, it is clear that they benefit from physical exercise programs if they are given the appropriate opportunity to participate in physical exercise [219,220,221]. They receive satisfaction from exercise, adopt healthy behaviors that are incompatible with substance use [44,77,214], and are accompanied by the subsequent activation of internal mechanisms that mobilize and motivate individuals to exercise more often. This eventually contributes to an active everyday life with rewarding benefits to their physical and mental health [222,223]. 

Thus, interventions aimed at creating an active environment by reducing sedentary time and increasing physical activity could have therapeutic potential for individuals receiving methadone treatment. This is confirmed by Colledge et al. [82], in which increased physical activity levels are a mobilizing factor leading to greater treatment adherence. At the same time, physical exercise is a low-cost intervention that fits the overall profile of a specific population [78,82,224]. It is considered a healthy and effective method of changing a bad mood and an authentic source of increasing pleasure and energy, which would otherwise be sought through artificial substitutes. In addition, many forms of exercise promote increased social inclusion and prevent loneliness and isolation, which have been associated with increased substance use [107]. Most importantly, exercise and participation in physical activities require internal activation. By discovering and activating their inner strength through exercise, patients will gradually realize the inner strength needed to fight and overcome their problems. 

The treatment plan for opioid replacement programs should include strategies that strengthen the patient’s change mobilization based on their potential. In this context, physical exercise can mobilize the patient and have positive therapeutic effects, such as the highest rates of abstinence from use [82]. The value of exercise as a motivating mechanism lies in emotional activations such as self-efficacy [115] as a result of improving the patient’s well-being and body image and helping them deal with their problem [82,224]. In addition, many forms of exercise can help patients integrate into social networks and adopt a healthy lifestyle that prevents loneliness and isolation, which is associated with increased substance use [107,115].

### 5.2. Exercise as a Self-Regulation and Management Factor 

There is growing evidence that opioids reduce reactivity to stressful stimuli by having anxiolytic or sedative effects [225,226]. Nonetheless, chronic use of opioids, whether prescribed or illicit, is associated with the development of long-term craving for the addictive substance [227]. Although the early stages of addiction focus on the “hedonic reward”, attention is also directed to the mechanism of “negative reinforcement”, i.e., the process where the withdrawal of an unpleasant stimulus (the negative emotional state of deprivation) increases the likelihood of a reaction (substance intake), which may have an essential role in retaining addictive behaviors [228]. Therefore, individuals with opioid use disorders (OUDs) have negative affect, distress, and craving. This can persist and contribute to higher levels of anxiety, which in turn may be a potential motivating factor for opioid use, particularly during periods of opioid withdrawal [229]. 

It becomes clear that stress is a major contributing factor to the onset of disorders in opioid users as a result of the passive way of dealing with everyday life [216,217]. Therefore, stress management is considered particularly important [230]. These people experience stress to a greater extent and are less likely to deal with it more effectively than the general population [44], resorting to substances for relief. On the other hand, physical exercise is positively related to a person’s ability to cope with daily stress without the use of substances [115,213]. Specifically, exercise causes “normal stress”, which in many cases regulates the external psychological stress as it can enhance communication between the biological axes that are activated when the person experiences stressful situations [210]. Indeed, exercise has been shown to reduce a person’s reactivity to stress [211] and can replace substance use as a more appropriately tailored intervention to address unpleasant and stressful situations [231]. 

Wang et al., [232] discovered that physical exercise not only increases abstinence rate from the aforementioned substances but also soothes symptoms of deprivation, anxiety, and depression in a meta-analysis on the effects of physical exercise on a state of dependence on different substances (alcohol, nicotine, opioids). Nevertheless, there is strong evidence that the effect of exercise varies depending on the type of addictive substance. Specifically, the effect of exercise is more beneficial in opioid addicts (morphine, heroin) than in alcohol and nicotine addicts. A possible explanation for that is the differences in addictive mechanisms for each of the addictive substances. For example, opioid drugs (morphine, heroin, etc.) act through the b-endorphin neurotransmitters and activate the opium receptors m and d [233]. Alcohol, on the other hand, promotes the GABAA receptor response [234], increasing dopamine and opioid receptor stimulation [235,236], while nicotine addiction is promoted by neurotransmitters activating the a2b4nACH receptor [237,238]. 

A pilot study in twenty-nine patients on maintenance therapy with methadone demonstrated that a video game-based intervention with physical activity, including a combination of aerobic and strength training exercises lasting a total of 25 min, resulting in a high acceptance rate by the participants. Moreover, they significantly reduced their use of cocaine and opioids and showed improved mental health, as anxiety and pessimism decreased. In addition, there was overall compliance among the participants in the detoxification program with the substitute substance. Furthermore, participants who completed the exercise program scored higher levels of physical activity outside of the supervised sessions compared to those who did not complete the program [83]. These findings suggest that physical exercise may prevent recrudescence of addictive behaviors, by helping individuals self-regulate their anxiety and mood, cope with emotional stress, build self-esteem and confidence, and achieve pleasurable situations without the use of substances [239]. 

Overall, exercise is recognized as a major, low-cost intervention in the treatment of opioid replacement programs. The research data supports the view that physical exercise can regulate stress and anxiety, remove accumulated tension, relieve a tense emotional state, reduce feelings of boredom and frustration, and relieve the symptoms of depression in these patients [115]. Moreover, exercise develops self-control, makes individuals more disciplined, cultivates restraint and self-regulation, and prevents the adoption or continuation of unhealthy behaviors.

### 5.3. Exercise as a Factor of Commitment and Adherence (Compliance)

According to clinical studies, exercise affects many variables related to the health and quality of life of patients on opioid replacement therapy [130,232]. Specifically, the results of these studies suggest that these patients benefit from participating in aerobic exercise programs and should therefore regularly exercise to enhance their therapeutic goal [93] and to treat symptoms for which opioid maintenance therapy appears to be deficient [96]. Indeed, physical exercise reduces the symptoms of pain [86], improves the quality of sleep [89,90], and is a key factor in maintaining the ideal body weight [88]. Similarly, observational studies also suggest exercise as an effective complementary intervention to improve chronic pain [240], anxiety, and depression [115,241], symptoms that are common among patients receiving methadone maintenance therapy [241]. 

The benefits and safety of exercise are supported by the study of Pérez-Moreno et al. [84], which evaluated an aerobic and resistance exercise training program in prisoners who were on methadone replacement therapy. After the completion of the 4-month training program, participants in the exercise group showed improved physical fitness, muscle strength, and overall quality of life compared to the control group. In addition, no side effects were reported by any participant in the exercise group, suggesting that physical exercise is safe even for patients under opioid agonist treatment in a hazardous environment. Moreover, the absence of side effects of exercise in this population is particularly important, as exercise can further offer these patients a multitude of beneficial effects, including anger management, mood regulation, the channeling of energy into acceptable activities, and relapse prevention [116]. 

In another study that evaluated relapse factors in both former and current heroin users, participants, among other interventions, considered exercise as an effective method for reducing the likelihood of relapse. More specifically, they experienced exercise as a pleasurable activity that increased discipline and facilitated the cessation of heroin [242]. The long-term benefits of exercise in patients receiving heroin-assisted treatment have been investigated in the study by Colledge et al. [82]. They reported that the group of patients that followed a high-intensity exercise training program exhibited overall higher compliance with the treatment protocol compared to the control group. However, regular exercise is also required for these patients to receive its beneficial effects, and the physical and mental health benefits are not maintained for a long time after the cessation of an exercise training program [243].

A recent systematic review found that patients on opioid maintenance therapy (OMT) can benefit from aerobic exercise programs. This is because aerobic exercise can reduce the symptoms of sleep disorders and pain sensitivity and prevent relapse to non-opioid use [50]. More specifically, it was shown that supervised aerobic exercise interventions were feasible and improved physical fitness, mental health (perceived anxiety and depressive symptoms), and quality of life of patients on OMT [82,83,84]. 

Finally, the study by Ding et al. [85] investigated the effectiveness of aerobic exercise as a complementary strategy for the prevention and treatment of osteoporosis in young women addicted to opioids. This study showed that exercise had a positive effect on the restoration of bone quality (Stiffness index, T scores, and G score). 

Overall, aerobic exercise appears to be effective in improving osteoporosis [38,39,85], chronic pain and hypersensitivity [86], sleep disorders [89,90], perceived anxiety [44], depression [13], weight gain [88] and poor quality of life [50] observed among patients receiving OMT. Moreover, physical exercise improves body image by increasing self-esteem and self-confidence [50]. These pleasant emotions induced by physical exercise reverse the negative attitude that characterizes the addicted person [115], making managing the emotional stress experienced during the therapeutic course much more comfortable. All these positive alterations increase the satisfaction feelings derived from physical exercise, resulting in its repletion and adoption [50], therefore, increasing the adherence of the addicted person to the therapeutic course [116].

## 6. A Conspectus 

Chronic opioid use is associated with structural and functional changes in the human brain that impair the balance between regulatory-prefrontal and cortical-subcortical circuits, thus leading to an increase in impulsive, immediate gratification behavior [54]. These alterations affect every stage of the addiction cycle as they (a) enhance the attractiveness and strengthen motivation for taking addictive substances [244], (b) reduce the sensitivity of pleasure-generating mechanisms during deprivation [245], (c) develop deprivation stress [183,246], (d) cause loss of the inhibition system’s controlling role [54] and (e) mediate habit consolidation via neuroanatomical pathways. More specifically, these neuroadaptive alterations include the reduction of the neurotransmitter dopamine in its D2 receptors [245], the reduction of the medial prefrontal cortex metabolism [247,248], as well as the increase in CRF levels in the amygdala during the withdrawal phase of addictive substances, which results in anxious behavior and an escalation of substance intake [183,246]. It is widely accepted that both healthy and clinical populations can significantly benefit from their participation in exercise training programs. However, in opioid-dependent patients, exercise results in multiple other positive adaptations. This is because, in parallel with the removal of cumulative stress and the discharge of their tense emotional state, exercise releases the feeling of thought blocking and reverses the negative attitude in these individuals. Thus, a person who is addicted to drugs and feels constant joy from exercise finds it easier to deal with emotional stress and personal crises and to stay in the therapeutic setting [116]. 

Therefore, in recent years, exercise interventions have been used adjunctively in the treatment of individuals with OUDs [69,71,72,100]. Neurobiological studies have revealed that regular physical exercise modifies neural circuits, such as the reward, inhibition, and stress systems [75,92,101], causing behavioral changes in these patients [69,73]. These findings support a model of sequential (temporal) effects of exercise in favor of gradual disengagement from addiction. The sequence by which the exercise-induced mechanisms are consolidated follows the pattern of Internal activation–Self-regulation–Commitment, eventually resulting in the modification of molecular and behavioral aspects of opioid addiction. 

Specifically, physical exercise exerts positive effects on the symptoms of addiction [69,74,75,91,92,98], acting first as an internal activation and motivation factor via its effects on the endogenous cannabinoid system, particularly by increasing its ligand, N-AEA [132], and also on the endogenous opioid system, thereby enhancing dopaminergic transmission [91]. Consequently, exercise increases the sensitivity of NAc function, which increases reward sensitivity. These changes mobilize the individual, inducing an internal activation that intensifies the physical and mental effort, and increases self-esteem and feelings of adequacy, which increase further the motivation for effort [110,111]. 

Then, exercise acts as a self-regulation and management factor through regulatory effects on the dopaminergic and glutamatergic systems [92]. In particular, by normalizing dopamine levels [139,142] and glutamate signaling, it reverses molecular lesions induced in the mPFC [98,180,181]. Consequently, it increases the sensitivity of mPFC function, which increases inhibitory control, executive function, and cognitive function, as well as emotion regulation. The changes form an active external environment that is internalized and assimilated by individuals [182]. 

Finally, physical exercise acts as a commitment and adherence-compliance factor, reducing cortisol levels through the recruitment of hippocampus-prefrontal-amygdala neurocircuitry, which leads to a reduction in responsiveness to stress stimuli [83,101,107]. This circuit restores homeostasis, may promote the management of emotion, and supports a healthy stress response and daily life management [101,136,209]. Despite the significant neurobiological benefits of exercise, a brain health screening is proposed as a standard of care to deal with possible comorbidities of reward dysregulation due to possible genetic causes and/or epigenetic insults [243]. Moreover, it should be mentioned that there are many premorbid precursors to addictive behavior in the pre-teen and teenage years. Exercise regimens cannot be sustained or maintained without identifying such premorbid indicators. Multiple types of attention, reading, hearing, memory disorders, organic brain injuries, and various educational types and interpersonal needs, in particular, tip pre-teens into the pre-addictive state before puberty This state includes smoking, early psychiatric disorders of low self-esteem, anxiety, and depression, as well as mood and sleep disorders [249]. Hence, attention should be paid to the early identification and treatment of such premorbid factors of addictive behavior to avoid the need to treat an opioid addict with various precursors, which is much more difficult than treating the opioid addict [249,250]. Regarding the psychological basis of addiction symptoms, physical exercise serves as an alternative strategy for managing and dealing with the effects of substance use disorders [83,106,107]. Based on the internal activation mechanism, the initial effect of exercise as a complementary intervention is the creation of a “stimulation environment” for opioid-dependent patients [82,222,223]. This effect increases their engagement in non-substance-related activities [83,115], leads to stress [115] and anxiety relief [13,251], improves physical health [83], and evokes pleasant emotions such as satisfaction and enjoyment [44,115,116]. In addition, many forms of exercise promote social inclusion and prevent the loneliness and isolation associated with increased substance use [107]. 

Based on the self-regulation mechanism, regular exercise functions as a regulating and managing factor for negative stimuli and emotions such as stress, anxiety, and depression [252]. People who exercise exhibit a better adaptation to stressful stimuli than those who do not exercise, due to a multitude of positive effects [115]. These exercise-induced effects include the prevention of relapse and the regulation of mental mood [116]. The opioid-dependent patients that exercise feel more attractive and have more confidence as they perceive their bodies as functional and strong [253]. Additionally, the increased physical strength and endurance make them able to perform many activities without discomfort or fatigue [254]. Overall, through the self-regulation mechanism, physical exercise can prevent relapse, help to manage stress and mood, cope with emotional stress, enhance self-esteem and self-confidence, and create pleasurable situations without substance use [239,255].

## 7. Conclusions

This review focuses on the possible mechanisms that mediate the beneficial effects of physical exercise in the treatment of OUDs. We also emphasize the sequential consolidation of these mechanisms and reveal that the neurobiological effects of exercise in combination with certain psychological mechanisms appear to promote its beneficial actions. People with OUDs report that enjoyment is a key feature of physical exercise during their treatment and early recovery. In this regard, physical exercise can be considered a complementary therapy that has the potential to address significant therapeutic deficits of opioid substitution therapy, such as chronic pain, sleep and mood disorders, and the difficulty of maintaining healthy body weight. Physical exercise forms a pleasant framework that supports opioid-dependent patients’ commitment to therapy and their efforts, while their way of life is positively affected by gradually adopting further health behaviors. These positive effects of exercise lead to an increase in self-efficacy and a sense of accomplishment in these patients, which in turn enhances their overall sense of self-efficacy in maintaining abstinence from addictive substances. As a take-home message, given all these favorable alterations induced by exercise both at the level of physical and mental health, exercise prescription should be recommended as a complementary therapy for patients on maintenance treatment.

## Data Availability

Not applicable.
